# Cineraria Maritima

**Published:** 1889-03

**Authors:** 


CINERARIA MARITIMA.—In *Notes on New Remedies* it is said reports have been published, based on communications from Dr. Mercer of Venezuela, of the peculiar potency of Cineraria Maritima in arresting and improving partial blindness induced by cataract of the eye. Dr. Mercer's observations are authentic and fairly conclusive, as his experiments were made on his own eyes, after a failure to find relief under the skill of a prominent London specialist. Ophthalmologists have long derided the possibility that the power of vision is sometimes resuscitated in the pupil after becoming extinct; that this is true, however, can no longer be denied, and a number of such cases were cited and published in 1887, by Dr. Paul Meyer, prompted in consequence of an inaugural address by Prof. Leber, of Goettingen (on spontaneous resorption of cataracta senilis). It is therefore not beyond possibility that such spontaneous resorption or recovery may be induced by the aid of remedial agents as Cineraria Maritima, and investigation, as soon as intelligently directed, may early realize such results.

In Venezuela the juice of the leaves of cineraria maritima is dropped into the pupil, two drops three times daily, continued for several months. The application causes no inflammation, and only a temporary burning sensation with lachrymal flow of short duration. The juice of the mature leaves is esteemed most efficacious.

